# The effect of comorbidities on diagnostic interval for lung cancer and mesothelioma: A cohort study using linked data from the Clinical Practice Research Datalink and the Cancer Registry

**DOI:** 10.23889/ijpds.v8i2.2241

**Published:** 2023-09-14

**Authors:** Imogen Rogers, Max Cooper, Anjum Memon, Elizabeth Ford

**Affiliations:** 1 Brighton and Sussex Medical School, Brighton, United Kingdom

## Objectives

Resilience in healthcare has been defined as “the capacity to adapt to challenges and changes at different system levels, to maintain high quality care”. This work aimed to investigate how the challenges posed by the presence of comorbidities impacted on the delivery of timely lung cancer/mesothelioma diagnosis in older patients.

## Methods

Patients with incident lung cancer/mesothelioma aged at least 65y in 2019 were identified in the Clinical Practice Research Datalink and linked Cancer Registry data. Diagnostic interval (DI) was defined as time from first presentation with a symptom suggestive of lung cancer/mesothelioma to diagnosis date, including symptoms up to 12 months pre-diagnosis. Co-morbidities were grouped as four “alternative explanation” conditions, which might mimic lung cancer symptoms, and ten “competing demand” conditions, which might delay cancer referral by competing for the clinician’s time. Other factors considered were usual consultation frequency, smoking and BMI. Associations with DI were investigated using multivariate linear regression.

## Results

Data were available for 10424 lung cancer/mesothelioma patients. In adjusted analyses DI was longer in patients with “alternative explanation” conditions, increasing by 27.6 (95%CI 22.9 – 32.4 days) and 72.0 (65.6, 78.4) days in patients with one and two or more conditions respectively. Number of competing demand conditions was not associated with DI in adjusted analyses. However, both usual consultation frequency and increasing consultation frequency in the year before diagnosis were independently positively associated with diagnostic interval, which was 23.0 (17.8, 28.3) days higher in patients with an increased consultation rate. DI was also increased in ever-smokers and in underweight patients compared to those in the normal weight range.

## Conclusion

The presence of conditions offering alternative explanations for lung cancer/mesothelioma symptom is associated with delayed diagnosis. Patients with higher consultation frequencies also had longer DIs, implying competing demand is also an issue. Strategies to increase the resilience of healthcare systems to these challenges to timely diagnosis should be considered.

